# Supplementary material for: Conditional chemoconnectomics (cCCTomics) as a strategy for efficient and conditional targeting of chemical transmission
Source: eLife. 2024 Apr 30;12:RP91927. doi: 10.7554/eLife.91927 (PMC11060718; doi:10.7554/eLife.91927)
Supplement: Figure 3—source data 3. [file elife-91927-fig3-data3.zip › Figure 3 source data3 part 1.zip!/Figure 3-Source data 3-part1/1071-1-CRISPResso_Report_osfbjvga.zip!/CRISPResso_on_1071_3/1a.Read_barplot.pdf]

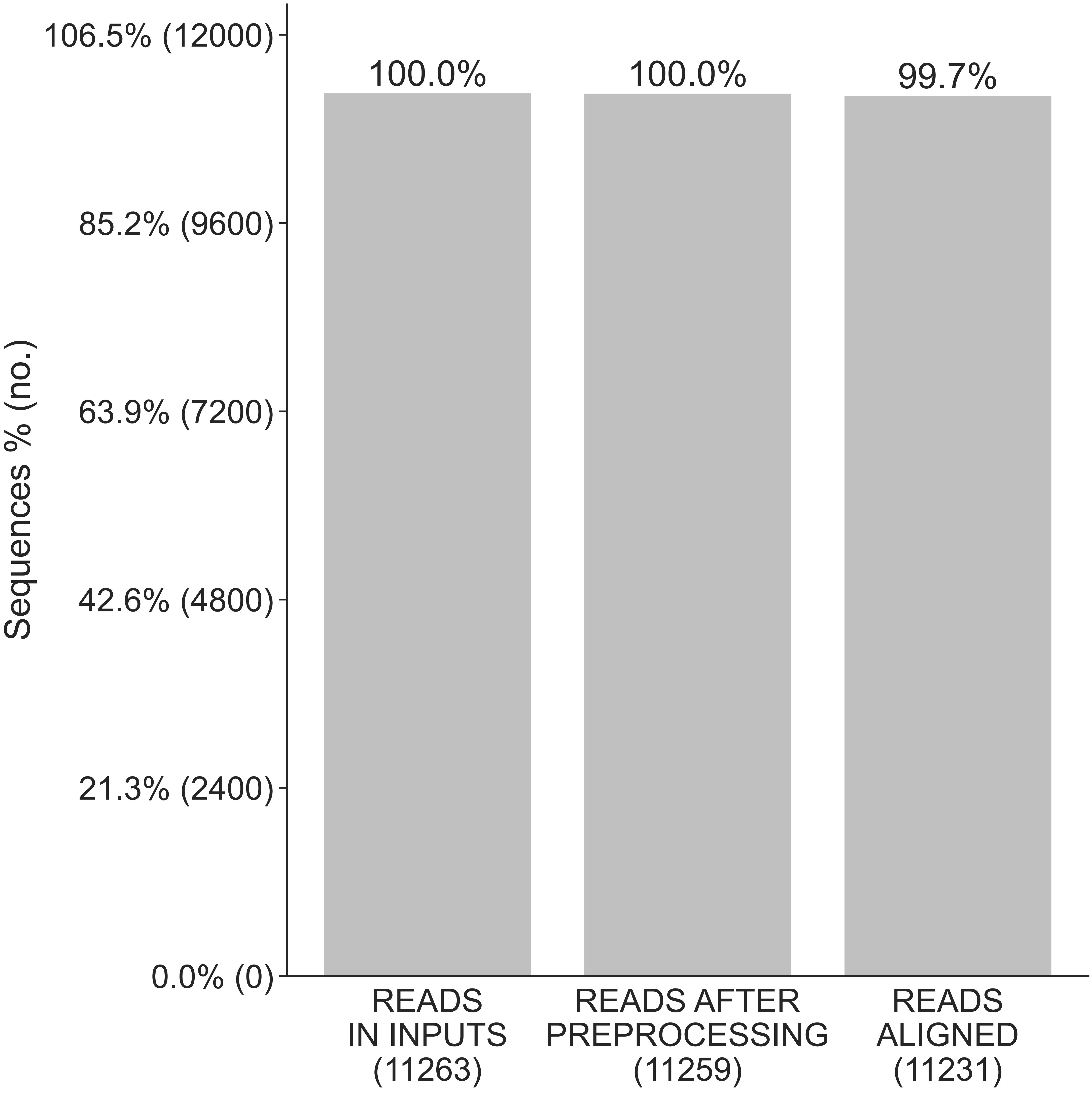

106.5% (12000)
85.2% (9600)
63.9% (7200)
42.6% (4800)
21.3% (2400)
0.0% (0)
Sequences % (no.)
100.0%
100.0%
99.7%
READS
IN INPUTS
(11263)
READS AFTER
PREPROCESSING
(11259)
READS
ALIGNED
(11231)
